# Acute neck pain caused by pseudogout attack of calcified cervical yellow ligament: a case report

**DOI:** 10.1186/s13256-016-0928-1

**Published:** 2016-05-30

**Authors:** Takashi Kobayashi, Naohisa Miyakoshi, Toshiki Abe, Eiji Abe, Kazuma Kikuchi, Hideaki Noguchi, Norikazu Konno, Yoichi Shimada

**Affiliations:** 1 Department of Orthopedic Surgery, Akita Kousei Medical Center, 1-1-1 Iijima-Nishifukuro, Akita, 011-0948 Japan; 2 Department of Orthopedic Surgery, Akita University Graduate School of Medicine, 1-1-1 Hondo, Akita, 010-8543 Japan; 3 Department of Orthopedic Surgery, Koto Kousei Hospital, 98-1 Kawasaki-Kaiho, Hachirogata-machi, Minamiakita-gun, Akita 018-1605 Japan

**Keywords:** Calcium pyrophosphate dihydrate, Yellow ligament, Acute neck pain, Pseudogout attack

## Abstract

**Background:**

Calcification of the yellow ligament sometimes compresses the spinal cord and can induce myelopathy. Usually, the calcification does not induce acute neck pain. We report a case of a patient with acute neck pain caused by calcium pyrophosphate dihydrate in a calcified cervical yellow ligament.

**Case presentation:**

A 70-year-old Japanese woman presented with acute neck pain. She had a moderately high fever (37.5 °C), and her neck pain was so severe that she could not move her neck in any direction. Computed tomography showed a high-density area between the C5 and C6 laminae suspicious for calcification of the yellow ligament. Magnetic resonance imaging showed intermediate-signal intensity on T1-weighted imaging and high-signal intensity on T2-weighted imaging surrounding a low-signal region on both T1- and T2-weighted imaging with cord compression. There was a turbid, yellow fluid collection in the yellow ligament at the time of operation. Histologically, calcium pyrophosphate dihydrate crystals were found in the fluid, and she was diagnosed as having a pseudogout attack of the yellow ligament.

**Conclusions:**

Pseudogout attack of the cervical yellow ligament is rare, but this clinical entity should be added to the differential diagnosis of acute neck pain, especially when calcification of the yellow ligament exists.

## Background

Calcification of the yellow ligament of the cervical spine is not rare and sometimes compresses the spinal cord and induces myelopathy [1]. Total removal of the yellow ligament is a treatment option. Macroscopically there are solid calcifications in the yellow ligament and pathologically calcium pyrophosphate dihydrate (CPPD) crystals are present [1].

Usually the calcification does not induce acute neck pain. However, CPPD deposition disease is known to induce acute arthritis in other joints such as the knee. We previously reported a series of patients with acute neck pain caused by lateral atlantoaxial joint CPPD crystal-induced arthritis [2]. However, to our knowledge, there are no reports of a histologically proven relationship between acute neck pain and CPPD crystal deposition in the yellow ligament of the cervical spine. We report a case of a patient with acute neck pain caused by CPPD in a calcified cervical yellow ligament.

## Case presentation

**Fig. 1:**
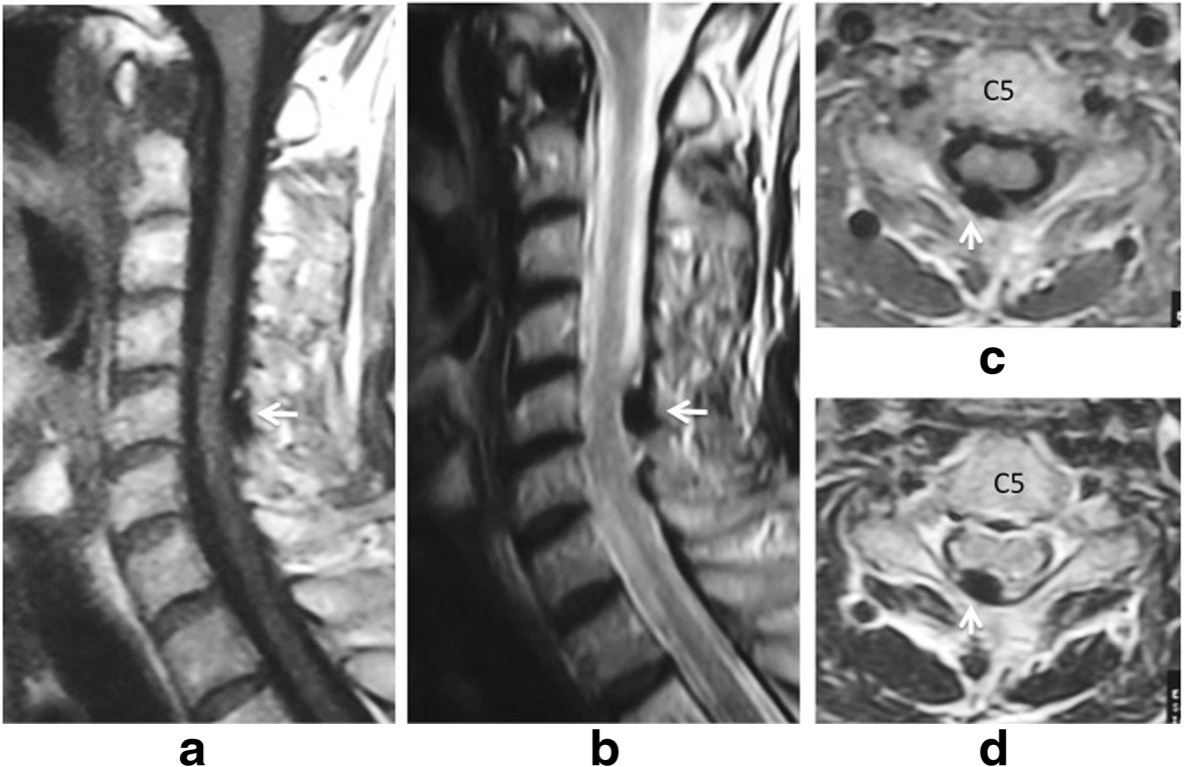
Sagittal T1-weighted imaging (**a**) and T2-weighted imaging (**b**), and axial T1-weighted imaging (**c**) and T2WI (**d**) magnetic resonance imaging on initial admission. Low signal intensity (*white arrow*) on both T1-weighted imaging (**a**, **c**) and T2-weighted imaging (**b**, **d**) at interlaminar space of C5-C6 and C6-C7 is seen on the sagittal image (**a**, **b**) and axial image (**c**, **d**)

**Fig. 2:**
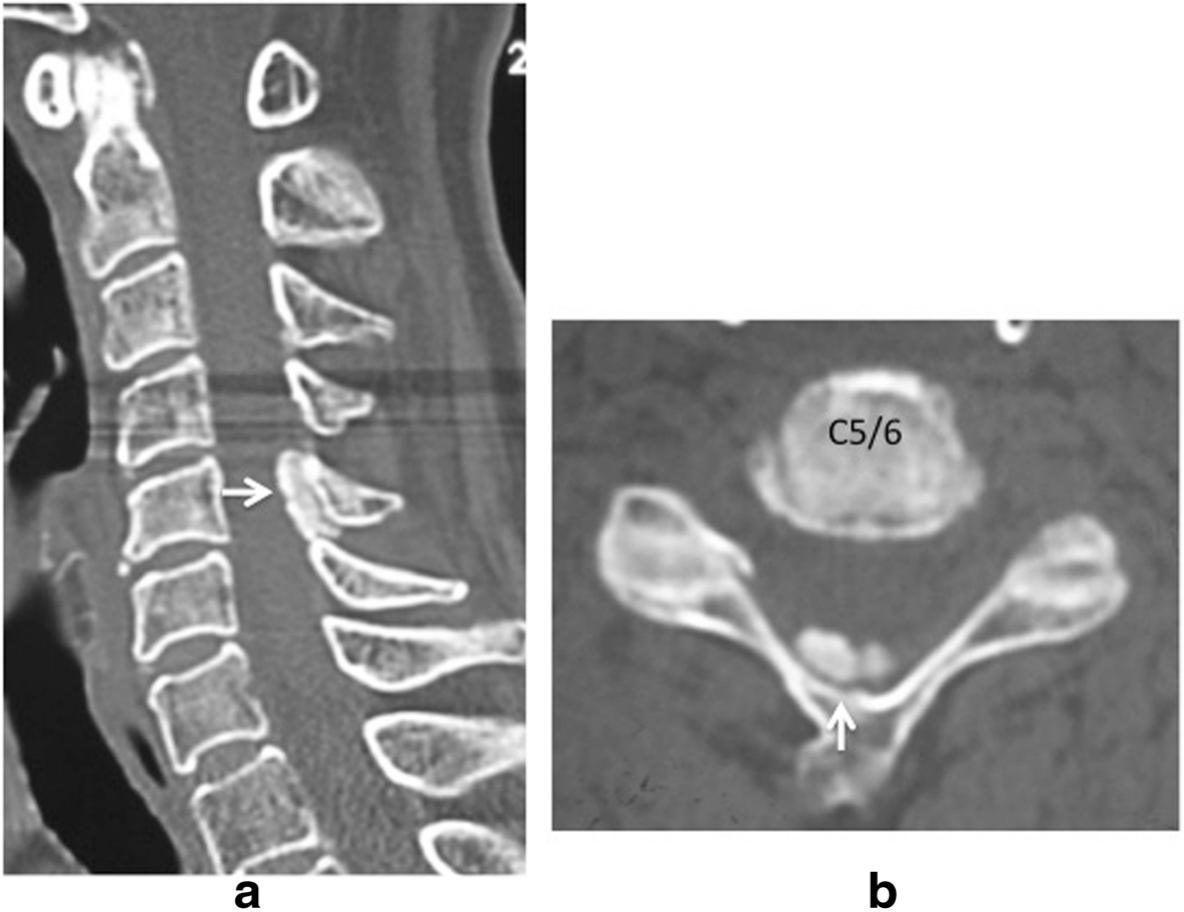
Computed tomography on the second admission. Amorphous high-density area at interlaminar space of C5-C6 (*white arrow*) is seen on sagittal image (**a**) and axial image (**b**)

**Fig. 3:**
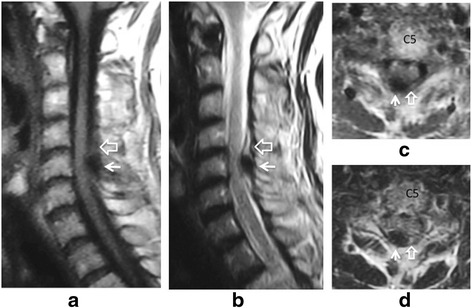
Sagittal T1-weighted imaging (**a**) and T2-weighted imaging (**b**), and axial T1-weighted imaging (**c**) and T2-weighted imaging (**d**) magnetic resonance imaging on the second admission. Low-signal intensity (*white arrow*) on both T1-weighted imaging (**a**, **c**) and T2-weighted imaging (**b**, **d**) surrounded by intermediate-signal on T1-weighted imaging and a high-signal intensity area on T2-weighted imaging (*open arrow*) at the interlaminar space of C5-C6 is seen on the sagittal image (**a**, **b**) and axial image (**c**, **d**). Axial images (**c**, **d**) show cord compression due to a low-signal intensity mass (*white arrow*)

**Fig. 4:**
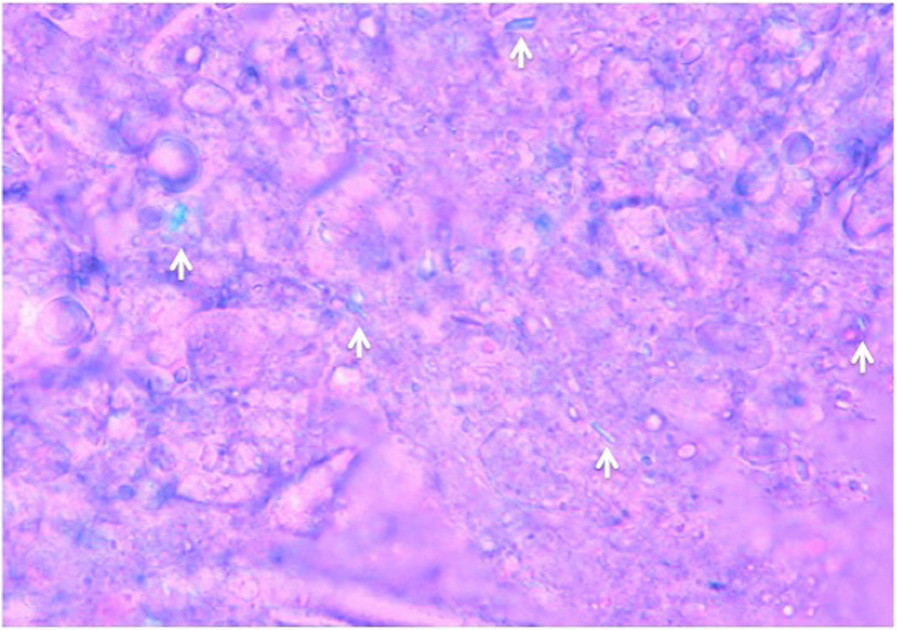
Polarized light microscopy of fluid in the yellow ligament. Various sizes of crystals with positive birefringence (*white arrows*) are seen. (Original magnification ×400)

A 70-year-old Japanese woman presented with a history of neck pain for 14 days before visiting our hospital. She had not been exposed to tuberculosis and had no history of recent head or neck injuries or diabetes mellitus. Her neck motion was slightly limited and she did not present with any neurological abnormality. Plain lateral X-ray showed calcification in the interlaminar space at C5-C6. Magnetic resonance imaging (MRI) showed low signal on both T1-weighted imaging (T1WI) and T2-weighted imaging (T2WI) (Fig. 1). Her neck pain was relieved gradually with nonsteroidal anti-inflammatory drugs (NSAIDs). Two months after her initial admission, her severe neck pain recurred. She had a moderately high fever (37.5 °C) on her second admission, and her neck pain was so severe that she could not move her neck in any direction. She did not complain of any joint pain suggesting arthritis. A physical examination revealed a severely limited range of motion of her neck in all directions. Her motor strength and sensation of her upper and lower extremities were unremarkable, and she had normal deep tendon reflexes. Her visual analog scale (VAS) pain score was 100 mm at her second visit, white blood cell count was 7800/mm^3^ (normal range, 3500–9300/mm^3^), and C-reactive protein was 5.13 mg/dL (normal range, 0–0.3 mg/dL). Computed tomography (CT) showed a high-density area between the C5 and C6 laminae with suspected calcification of the yellow ligament (Fig. 2). MRI showed intermediate-signal intensity on T1WI, and high-signal intensity on T2WI surrounding a low-signal region on both T1WI and T2WI with cord compression (Fig. 3), suggesting pseudogout attack or epidural abscess. Although NSAIDs were administrated, her neck pain persisted. Surgical decompression with C6 laminectomy and removal of the C5-C6 yellow ligament was performed 2 weeks after her second admission. There was a turbid, yellow fluid collection in the yellow ligament at the time of operation. There were visible calcifications in the yellow ligament, and dura adhered to the yellow ligament. Histologically, CPPD crystals were found in the fluid (Fig. 4), and she was diagnosed with a pseudogout attack of the yellow ligament. Our patient’s postoperative course was unremarkable. Her neck pain decreased, and laboratory data normalized 3 weeks after the operation.

Six months postoperatively she had no further severe neck pain. At a follow-up evaluation 7 years after initial onset, our patient had complete relief of neck pain, and there were no neurological abnormalities.

## Discussion

This patient’s course highlights two important clinical issues. First, pseudogout attacks of the cervical yellow ligament can cause acute neck pain. Second, calcification of the cervical yellow ligament raises suspicion for pseudogout.

There have been some reports of cervical canal stenosis caused by CPPD crystal deposition [1]. Myelopathy caused by nodular deposition of CPPD crystals in the yellow ligament of the cervical spine have been occasionally reported [1]. However, there are no known reports of histologically proven pseudogout attack of the cervical yellow ligament. Acute neck pain is often caused by crystal-induced arthritis of the lateral atlantoaxial joint [2] or by crowned dens syndrome (CDS) [3] in the elderly. For the diagnosis of crystal-induced arthritis, it is very important to identify calcium pyrophosphate crystals in the joint fluid. There was one report stating that lumbago originates from the presence of CPPD crystals in the vertebrae. Fujishiro reported a case of pseudogout attack of the lumbar facet joint in which CPPD crystals were found in the drained fluid [4]. In addition, we previously reported cases of pseudogout attack of the lateral atlantoaxial joint [2]. We found CPPD crystals in the fluid drained from the atlantoaxial joint. In the present case, CPPD crystals were found in the yellow ligament, and symptoms were relieved by removal of the yellow ligament. There is a close relationship between facet cysts and the yellow ligament. It is known that facet arthrography enhances cysts of the yellow ligament. In our case, we did not perform cervical facet arthrography. At surgery, when the yellow ligament was detached from the laminae, turbid fluid was released, and we suspected there were CPPD crystals in the yellow ligament. It is not clear whether the CPPD crystals were from the facet joint, but at surgery, the facet joint was not swollen, suggesting no facet arthritis.

For diagnosis CT and MRI are important. On CT, calcification of the yellow ligament was seen in the present patient. MRI is the preferred imaging method because of the excellent soft tissue contrast that is achievable. In this case, effusion around the calcification was visible. Surgery is needed if myelopathy is present. It is possible that this patient may have been treated without surgery because cord compression was not very severe. Conservative treatment with NSAIDs or cortisone is another treatment option. However, surgical decompression is a safe and effective treatment for calcification of the cervical yellow ligament. In this case, surgical decompression was chosen because the symptoms remained after conservative treatment.

Pseudogout attack usually occurs in a joint with radiographically proven calcium deposition. Although pseudogout can occur without radiographic evidence of chondrocalcinosis [5, 6] radiographic calcium deposition is a raise suspicion for pseudogout attack. In the spine, Finckh reported that, at the atlas, transverse ligament calcification is associated with the development of neck pain [7]. We previously reported that calcification of the transverse ligament may raise suspicion for atlantoaxial pseudogout [2]. Imai and Hukuda reported cases with attendant granulation tissue in the cervical yellow ligament in patients who experienced recurrent attacks of neck pain and fever with coincidental radicular pain in the upper arm [8]. Imai and Hukuda reported three cases of recurrent neck pain, but the cause of neck pain was not documented. Our case is the first report of neck pain caused by calcification of the yellow ligament, which was proved by both MRI and histological findings.

## Conclusions

Pseudogout attack of the cervical yellow ligament is rare but this clinical entity should be added to the differential diagnosis of acute neck pain, especially when calcification of the yellow ligament is present.

## Abbreviations

CDS, crowned dens syndrome; CPPD, calcium pyrophosphate dihydrate; CT, computed tomography; MRI, magnetic resonance imaging; NSAIDs, nonsteroidal anti-inflammatory drugs; T1WI, T1-weighted imaging;T2WI, T2-weighted imaging; VAS, visual analog scale.
